# A novel sucrose transporter gene *IbSUT4* involves in plant growth and response to abiotic stress through the ABF-dependent ABA signaling pathway in Sweetpotato

**DOI:** 10.1186/s12870-020-02382-8

**Published:** 2020-04-15

**Authors:** Dandan Wang, Hongjuan Liu, Hongxia Wang, Peng Zhang, Chunyu Shi

**Affiliations:** 1 State Key Laboratory of Crop Biology, College of Agronomic Science, Shandong Agricultural University, Tai’ an, 271018 China; 2 National Key of Plant Molecular Genetics, CAS Center for Excellence in Molecular Plant Sciences, Institute of Plant Physiology and Ecology, Chinese Academy of sciences, Shanghai, 200032 China

**Keywords:** Sweetpotato, Sucrose transporters, Plant growth, Abiotic stress, ABA signaling pathway

## Abstract

**Background:**

To maintain sweetpotato (*Ipomoea batatas* (L.) Lam) growth and yield, sucrose must be transported from the leaves to the roots. Sucrose transporters or carriers (SUTs or SUCs) transport sucrose and are involved in plant growth and response to abiotic stress. However, the mechanisms of SUTs in sweetpotato abiotic stress resistance remains to be determined.

**Results:**

In the present study, we cloned a novel *IbSUT4* gene; the protein encoded by this gene is localized in the tonoplast and plasma membrane. The plant growth was promoted in the *IbSUT4* transgenic *Arabidopsis thaliana* lines, with increased expression of *AtFT*, a regulator of flowering time in plants. Over-expression of *IbSUT4* in *Arabidopsis thaliana* resulted in higher sucrose content in the roots and lower sucrose content in the leaves, as compared to the wild-type (WT) plants, leading to improved stress tolerance during seedling growth. Moreover, we systematically analyzed the mechanisms of *IbSUT4* in response to abiotic stress. The results suggest that the ABRE-motif was localized in the *IbSUT4* promoter region, and the expression of the ABA signaling pathway genes (i.e., *ABF2*, *ABF4*, *SnRK2.2*, *SnRK2.3,* and *PYL8/RCAR3*) were induced, and the expression of *ABI1* was inhibited.

**Conclusions:**

Our dates provide evidence that *IbSUT4* is not only involved in plant growth but also is an important positive regulator in plant stress tolerance through the ABF-dependent ABA signaling pathway.

## Background

In higher plants, photoassimilates are transported from source organs to sink organs mainly in the form of sucrose. The synthesis, storage, transportation, and utilization of sucrose are some of the major determinants of the cell activity, organ and tissue development, and crop yield [1]. Sucrose transportation is mediated by various proteins, of which sucrose transporters or carriers (called SUTs or SUCs) mediate sucrose movement across membranes.

Plant SUTs belong to the major facilitator superfamily (MFS) and are integral membrane proteins with 12 transmembrane-spanning regions [2]. SUTs are assumed to be H^+^-sucrose transporters involved in cellular proton-coupled sucrose uptake and may serve two main functions in the apoplastic pathway: loading sucrose into the phloem in source leaves and unloading sucrose into the cells of sink organs, such as the storage roots, tubers, fruits, and developing tissues [3]. Several SUTs have been identified and characterized in plants. For example, Riesmeier et al. (1992) first cloned spinach SUT cDNA and verified its transport function in yeast [4]. Subsequently, the SUTs of *Arabidopsis thaliana* were reported. Sauer and Stolz (1994) used spinach *SUT* cDNA as a probe to screen the *Arabidopsis thaliana* cDNA library, isolating *SUT1* and *SUT2* [5]. The *AtSUC*s were analyzed and found to consist of three distinct subfamilies. The low substrate specificity protein encoded by *AtSUC2* is highly expressed in the collection phloem, and is involved in phloem loading and the retrieval of leaked sucrose in the transport phloem [6, 7]. In the absence of sucrose, *AtSUC2* mutant seedlings were smaller than wild-type (WT) seedlings [8]. Compared to other SUCs, *AtSUC9* has an ultrahigh affinity for sucrose. Plants with *AtSUC9* mutations are sensitive to low sucrose levels and have an early flowering phenotype under short-day conditions, suggesting that *AtSUC9* is associated with floral induction [9]. In contrast to *AtSUC9*, *AtSUC4*, which belongs to the SUT4 clade, has low sucrose affinity and is the only *Arabidopsis* SUC known to be localized in the tonoplast, releasing sucrose from the vacuole [10]. In addition to *Arabidopsis thaliana*, SUCs in other plants have also been studied. Inhibition of the *StSUT4*, mainly expressed in sink organs such as tubers, sink leaves, and flowers, leads to early flowering, increased tuber yield, and decreased in sensitivity toward a far-red light, which may be owing to increased sucrose export from the leaves at the end of the light period [11]. Transgenic poplars with RNAi-suppressed *PtaSUT4* had increased leaf-to-stem biomass ratios, elevated sucrose in the source leaves and stems, and altered carbohydrate-active enzymes [12]. Together, these studies demonstrate that SUTs are involved in the carbohydrate partitioning of source and sink organs.

SUTs are also involved in the plant’s response to various abiotic stressors; environmental factors impact the source-sink relationship, carbon allocation, and plant growth [1]. The loss-of-function mutation in *AtSUC2* and *AtSUC4* leads to hypersensitivity to drought, salt, and cold stress, while abscisic acid (ABA) treatment during seed germination and seedling growth lead to high sucrose content in the shoots but low sucrose content in the roots [13]. Compared to WT *Populus* plants, in those silenced for *SUT4,* acute drought conditions resulted in reduced water uptake rates and delayed wilting [14]. A recent global analysis of publicly available gene expression data was conducted to assess the regulation of SUT genes in response to different environmental stimuli [15]. The results indicated that SUTs respond to abiotic stressors, but the stress-induced effects on the sucrose distribution and the underlying molecular mechanisms remain unexplored.

ABA is a broad-spectrum phytohormone involved in the regulation of stomatal opening, growth and development, and coordination of various stress signal transduction pathways during abiotic stress [16, 17]. To date, several ABA-pathway genes responsible for plant tolerance to abiotic stress have been identified, including the ABA receptor *PYL8/RCAR3* and the regulatory factors SNF1 (SNF1-activated protein kinase) and PP2C (2C protein phosphatases) [18–21]. Moreover, it has been reported that ABA is associated with the sugar response pathway in plants [22]. Jia et al. (2015) found that low sucrose levels induced *AtSUC9*, which accumulates ABA through the ABA-pathway ABA-inducible genes, to enhance abiotic stress resistance [23].

Sweetpotato is a dicotyledonous crop, with underground storage organs. Its yield is closely related to the transport and distribution of photosynthetic products. The sweetpotato SUT genes *IbSUT1* and *IbSUT2* were previously isolated, and an immunolocalization study found that *IbSUT2* is localized in the SE-CCs (sieve element-companion cells) [24, 25]. However, the characteristics and functions of the sweetpotato SUTs and the mechanisms by which they mediate sucrose transport to support the growth and development of sink organs in response to environmental stimuli are unknown. Here, we report the double-membrane localization of a novel sweetpotato SUT gene, *IbSUT4*, and demonstrate its involvement in the abiotic stress response through the ABF-dependent ABA signaling pathway.

## Results

### *IbSUT4* encodes a conserved SUT protein and is homologous to *Solanum StSUT4*

To investigate the functions of the sweetpotato SUTs, we cloned a gene (Gene Bank: MN233360) and named it *IbSUT4*. The expected 463 bp EST fragment was amplified using degenerate primers SpSUT4-F and SpSUT4-R (Fig. S1A). The specific primers were designed according to the obtained EST sequences from the amplification of 3′ and 5′ cDNA, and the lengths of the 3′ and 5′ends of *IbSUT4* were obtained (Fig. S1B). The primers were generated by splicing the sequences, and the entire 1623 bp sequence was obtained, with the longest open reading from (ORF) of 1512 bp.

**Fig. 1:**
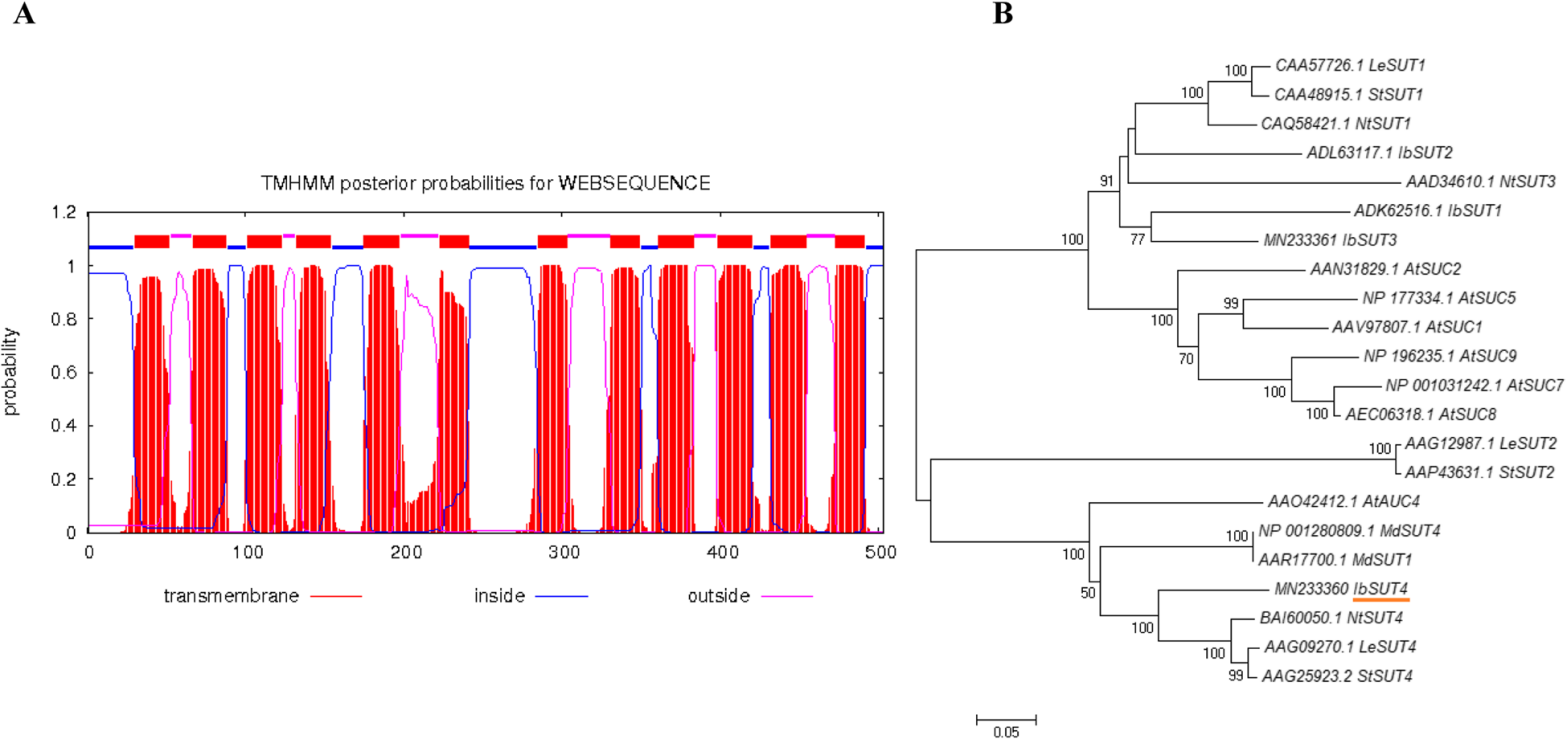
Analyses of *IbSUT4.***a**. Hydrophilicity and hydrophobicity analyses of the predicted amino acid sequence encoded by *IbSUT4.***b**. Phylogenetic analysis of the IbSUT4 and SUTs proteins from other plant species. The bootstrap consensus tree from 1000 replicates was constructed based on the neighbor-joining method, using MEGA 5.2

Quantitative reverse transcription PCR (qRT-PCR) indicated that *IbSUT4* was expressed at very high levels throughout the plant, with relatively higher levels in the sink organs (Fig. S1C). Protein prediction software revealed that the IbSUT4 protein was 502 amino acids long and contained one MFS domain and 12 trans-membrane regions, which is a typical feature of MFS family (Fig. 1a). The MFS family as a membrane transport protein, whose basic function is to assist trans-membrane transport [26].

To explore the relationship between the IbSUT4 protein and other plant SUTs, phylogenetic analyses were performed with the amino acid sequences. BlastP and phylogenetic analyses showed that the protein encoded by *IbSUT4* belongs to the MFS family. Furthermore, *IbSUT4* belonged to clade IV and was a close homolog of *StSUT4* and *AtSUT4*, both of which are involved in the abiotic stress response (Fig. 1b). This indicates that *IbSUT4* may also play an important role in the response to abiotic stress.

### *IbSUT4* localizes to the tonoplast and plasma membrane

**Fig. 2:**
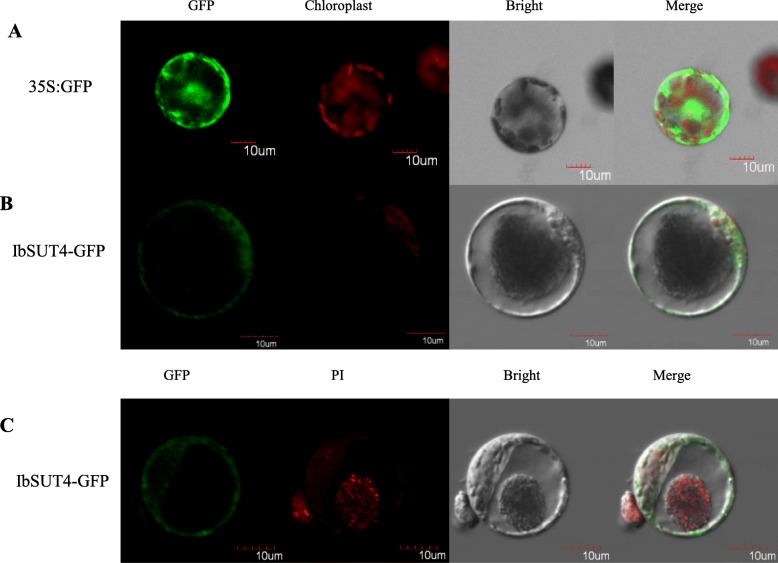
IbSUT4 localized to the tonoplast and plasma membrane. **a**, **b** Protoplasts expressing different GFP-fusion constructs. **c** Protoplasts expressing the *IbSUT4*-GFP-fusion construct incubated with 50 μg mL^− 1^ PI in W5 buffer (pH = 5.7) for 30 min. GFP is indicated in green, and PI fluorescence and chlorophyll autofluorescence are indicated in red. A. Overview image of the tobacco protoplasts transformed with p35S-GFP. B. Overview image of tobacco protoplasts transformed with p35S:*IbSUT4*-GFP. C. Protoplasts expressing p35S:*IbSUT4*-GFP with PI in the nucleus. Scale bar =10 μm

Based on the subcellular localization of *StSUT4* [11, 27], we predicted that *IbSUT4* would also be localized in the tonoplast and plasma membrane. We constructed a C-terminal translational fusion of GFP to *IbSUT4*, under the control of the Cauliflower Mosaic Virus 35S promoter (CaMV35S:IbSUT4-GFP). The GFP signal of the fusion protein was unevenly distributed along the membrane in tobacco protoplasts (Fig. 2). The irregular and rounded distribution of GFP suggested that *IbSUT4* may be associated with the vacuolar membrane of tobacco protoplasts.

To test if *IbSUT4* was localized in the tonoplast, we used Propidium Iodide (PI) to dye the nucleus. The nucleus was stained red, and the GFP signal was detected around the nucleus (Fig. 2). These results suggest that *IbSUT4* is localized to the tonoplast and plasma membrane.

### *IbSUT4* encodes a functional sucrose transporter

**Fig. 3:**
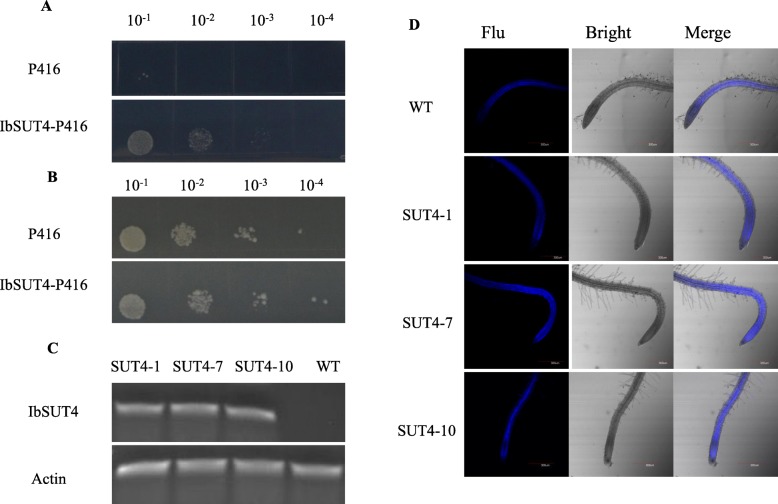
*IbSUT4* function analysis. **a**, **b** Expression of *IbSUT4* in the SUSY7/ura3 yeast strain grown on sucrose or on glucose as the sole carbon source. The plates were incubated at 30 °C for 3 days before observation. A. SUSY7/ura3 yeast transformed with the empty P416 vector and with *IbSUT4* in the P416 vector, and grown on 2% sucrose. B. SUSY7/ura3 yeast transformed with the empty P416 vector and with *IbSUT4* in the P416 vector, and grown on 2% glucose. **c**, **d***IbSUT4* shows sucrose uptake activity in *Arabidopsis thaliana*. C. RT-PCR analysis of *IbSUT4* expression in the WT and three transgenic lines (*SUT4*–1, *SUT4*–7, and *SUT4*–10). D. Uptake of fluorescent esculin in seven-day-old seedlings. **a**, **b**, **c** The blots/gels are cropped from Additional file 2, Fig. S.2,3. Scale bar = 300 μm

To verify the sucrose uptake activity of the sweetpotato SUT, we diluted the yeast solution and coated the plate. On media containing 2% sucrose as the sole carbon source, SUSY7/ura3 transformed with *IbSUT4* grew better than yeast transformed with an empty p416GPD vector (Fig. 3a). However, on media containing 2% glucose as the sole carbon source, SUSY7/ura3 transformed with *IbSUT4* and the empty p416GPD vector did not differ (Fig. 3b). The expression of *IbSUT4* allowed yeast to grow on sucrose, indicating that *IbSUT4* encodes a functional SUT.

To observe the activity of *IbSUT4* in vivo, an expression vector 35S:*IbSUT4* was constructed and introduced into *Arabidopsis*, resulting in six homozygous transgenic lines, resistant to hygromycin. Semi-quantitative RT-PCRs of the transcript levels of *IbSUT4* in the transgenic lines showed that *IbSUT4* was up-regulated in *Arabidopsis thaliana* (Fig. 3c). We selected *SUT4*–1, *SUT4*–7, and *SUT4*–10 for further analyses. We used esculin (6, 7-dihydroxycoumarinβ-D-glucoside), the fluorescent sucrose analog of sucrose, to observe the transcription activity in *Arabidopsis thaliana*. WT and the three transgenic lines were grown on 1/2 MS medium for 7 days. The plants were then incubated for 90 min in a 1/2 MS liquid solution containing 1 mM esculin, and the blue fluorescent signal intensity was observed. The signal intensity in the roots of transgenic *Arabidopsis thaliana* was significantly stronger than in WT, indicating the intracellular absorption and accumulation of esculin in the roots (Fig. 3d). To increase the accuracy and reliability of the results, the fluorescence intensity was quantified in Image J, and the results reconfirmed previous observations (Fig. S2), implying that *IbSUT4* showed sucrose uptake activity, and this activity was enhanced in *IbSUT4-*expressing lines.

### Over-expression of *IbSUT4* results in early flowering in *Arabidopsis thaliana*

**Fig. 4:**
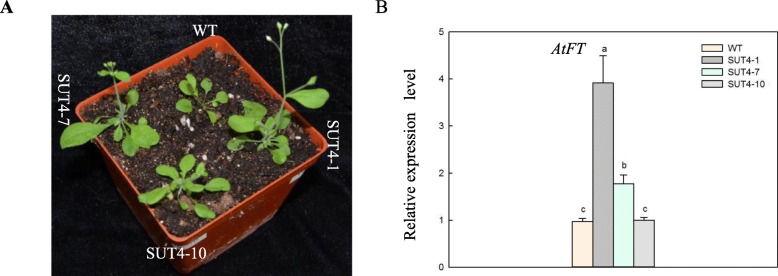
*IbSUT4* is involved in the plant growth. **a**. The three *IbSUT4* transgenic lines (*SUT4*–1, *SUT4*–7, and *SUT4*–10) showed earlier flowering than the WT when grown under LD conditions. **b**. The expression of the early flowering marker gene *AtFT1*. Lowercase letters indicate statistically significant differences (*P* ≤ 0.05)

After 13 days of growth under 16 h light / 8 h dark (long day, LD) conditions, we assessed the number of flowering plants (Table S2) and found that the *IbSUT4-*expressing *Arabidopsis thaliana* flowered earlier and had larger leaves than the WT plants (Fig. 4a). We also quantified the expression of the flowering marker gene, *AtFT*. The *FLOWERING LOCUS T* (*FT*) gene is the key locus determining the flowering time of plants through photoperiodic pathway. The *FT* gene also plays a role in the regulation of vegetative growth and morphogenesis in plant organs [28]. The expression of *AtFT* differed significantly between the WT and three transgenic *Arabidopsis thaliana* lines (Fig. 4b), indicating that *IbSUT4* expression induced early flowering. These results suggest that *IbSUT4* is involved in plant growth.

### Over-expression of *IbSUT4* improves sensitivity to ABA during seed germination and increases stress resistance during seedling growth

To gain insights into the possible functions of *IbSUT4*, abiotic stress (low temperature, mannitol, and high salt) and ABA treatments were performed in sweetpotato. The qRT-PCR results suggested that *IbSUT4* responds to multiple stressors (Fig. S3), based on these results, we selected high salt, low temperature, and ABA stress to investigate *IbSUT4* expression in *Arabidopsis thaliana*.

**Fig. 5:**
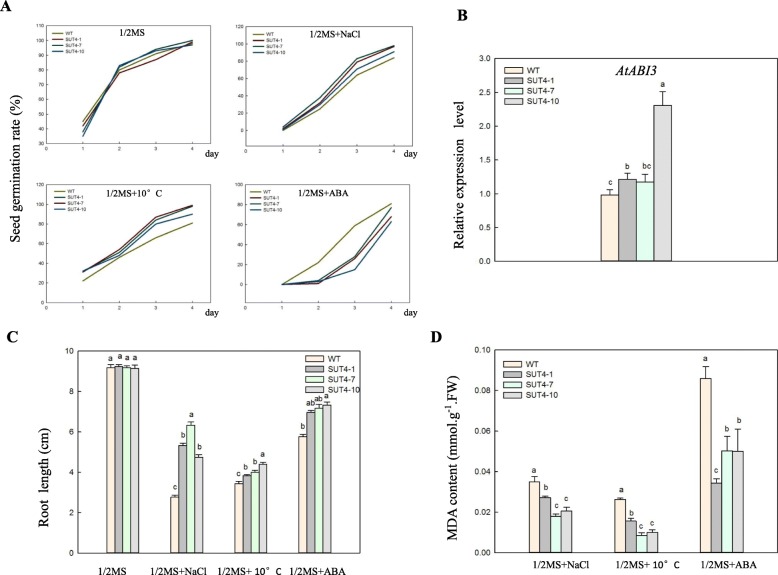
Seed germination and seedling growth in the WT and the three *IbSUT4* transgenic lines (*SUT4*–1, *SUT4*–7, and *SUT4*–10) under abiotic stress conditions. **a**. The germination rates were recorded in 1/2 MS media with or without NaCl, 10 °C, or ABA treatments during a period of 1 to 4 d after stratification. **b**. The expression of *AtABI3* in seeds under the ABA treatment for 4 d. **c** and **d**. 14 day-old seedlings grown with or without NaCl, 10 °C, or ABA were analyzed for their root length and MDA content. Lowercase letters indicate statistically significant differences (*P* ≤ 0.05)

Under control conditions, the rate of seed germination rates in the WT and transgenic lines did not differ. Under low temperature and high salt stress conditions, the germination rates were also similar. Interestingly, the germination rates of the transgenic lines were significantly suppressed by treatment with 1 μM ABA (Fig. 5a). To further investigate this phenomenon, we analyzed the *AtABI3* gene by qRT-PCR. ABA and GA hormones play an important role in dormancy and germination [29, 30], and AtABI3, an ABA response factor, has a negative regulatory role in seed germination that can inhibit seed germination at high expression levels [31]. *AtABI3* expression was significantly higher in the transgenic lines than in WT under 1 μM ABA stress (Fig. 5b), suggesting that over-expression of *IbSUT4* resulted in improved sensitivity to exogenous ABA during seed germination.

We further investigated the effects of abiotic stress on seedling growth by examining the roots lengths. There were no significant differences in roots growth between WT and transgenic lines under control conditions, but the transgenic seedlings exhibited longer roots under abiotic stress (Fig. 5c). Quantitative analyses showed that the roots of the transgenic seedlings produced less malondialdehye (MDA) (Fig. 5d). These results suggest that over-expressing of *IbSUT4* can improve plant resistance to abiotic stress.

### Over-expression of *IbSUT4* alters the sucrose distribution in shoots and roots in response to salt, low temperature, and exogenous ABA treatments in *Arabidopsis thaliana*

**Fig. 6:**
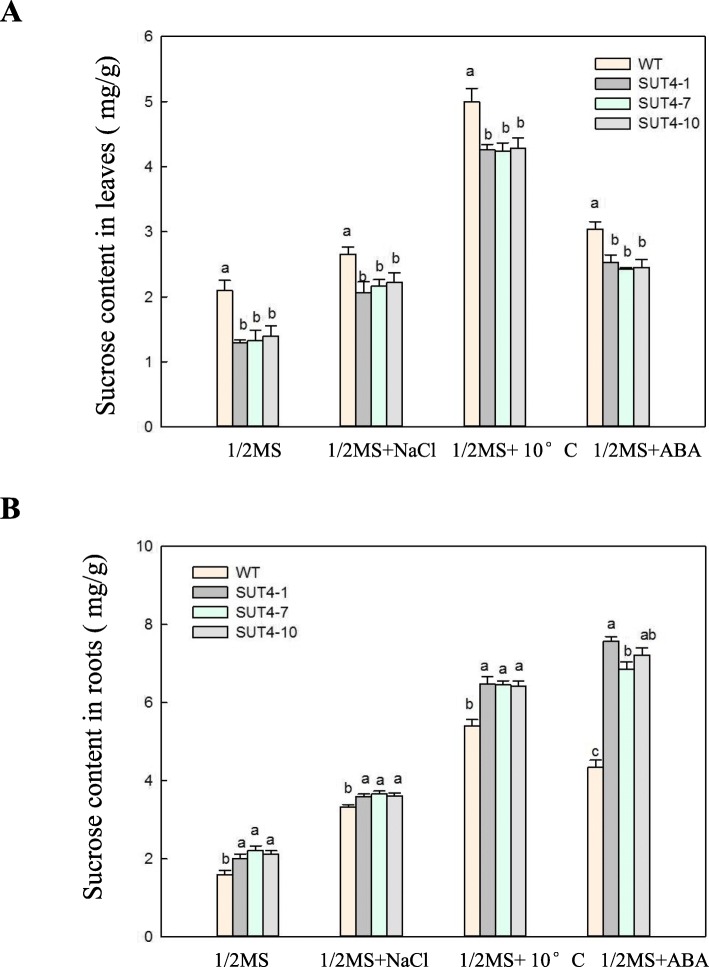
The sucrose distribution was changed in *IbSUT4* transgenic *Arabidopsis thaliana* lines under control and abiotic stress conditions, including low temperature (10 °C), high salt (100 mM NaCl), and exogenous 10 μM ABA treatments. The sucrose content of the shoots (**a**) and roots (**b**) of 14 day-old WT and transgenic seedlings under abiotic stress and ABA treatments. Lowercase letters indicate statistically significant differences (*P* ≤ 0.05)

Previous studies have shown that *AtSUC4* mutants exhibit altered sucrose distributions in the shoots and roots during stress [13]. Therefore, we measured the sucrose content of WT and transgenic lines under stress conditions. The roots of the transgenic lines exhibited greater accumulation of sucrose than the leaves (Fig. 6), suggesting that *IbSUT4* is required for sucrose distribution to the roots under stress conditions. Interestingly, the sucrose contents of the WT and transgenic roots was higher under various stress conditions than under the control traetment, suggesting that sucrose accumulation in the roots may be important for plant stress tolerance. These results show that the sucrose distribution is altered in transgenic lines during stress, which may explain better transgenic plant growth under stress conditions.

### Over-expression of *IbSUT4* induces the expression of ABA signaling pathway genes

To explore the molecular mechanisms of the plant stress response, we obtained the 847 bp promoter of *IbSUT4* by genome walking. Analyses using the plant CARE software revealed several stress and hormone response elements, including MYB, LTR, ABRE, and AuxRR-core (Table S3). The ABRE response element was also in the *IbSUT4* promoter region. The ABRE-binding transcription factors plays an important role in ABA signaling [32, 33], so we speculate that the over-expressed *IbSUT4* lines respond to stress via ABA signaling.

**Fig. 7:**
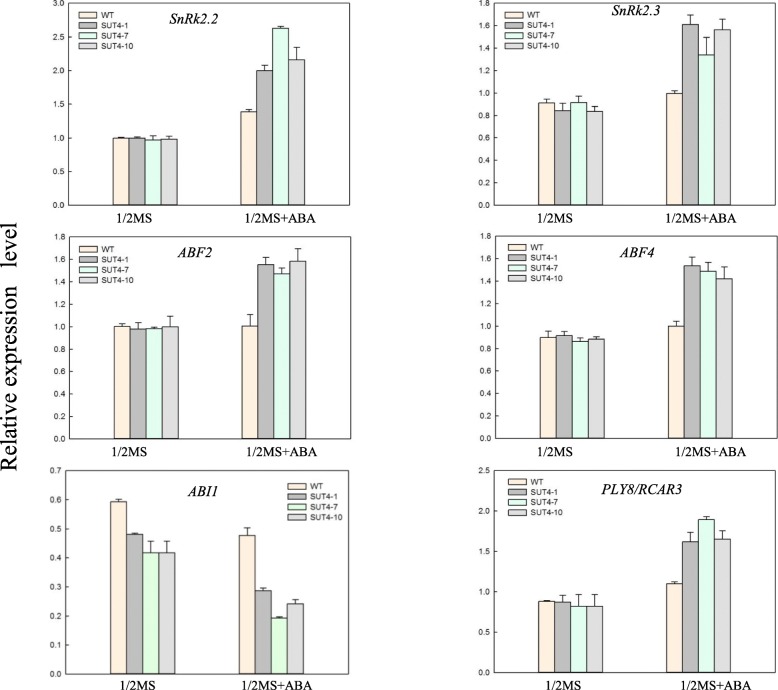
Over-expression of *IbSUT4* enhances the expression of the ABA signaling pathway genes. Gene expression was assessed by qRT–PCR in the WT and three *IbSUT4* transgenic *Arabidopsis thaliana* lines (*SUT4*–1, *SUT4*–7, and *SUT4*–10) in 1/2 MS, with no ABA treatment or 1/2 MS + ABA treatment with 40 μM ABA for 6 h. Lowercase letters indicate statistically significant differences (*P* ≤ 0.05)

We further verified the plants response to stress through the ABF-dependent ABA pathway by quantifying the expression of the ABA signaling pathway genes including *ABF2*, *ABF4*, *SnRK2.2*, *SnRK2.3*, *PYL8/RCAR3* and *ABI1* with qRT-PCR. In the absence of ABA, the expression of *SnRK2.2*, *SnRK2.3*, *ABF2*, *ABF4,* and *PYL8/RCAR3* did not obviously differ between the WT and three transgenic lines*.* Treatment with 40 μM ABA for 6 h resulted in higher expression for most of the genes in the transgenic lines than in the WT, except *ABI1* (Fig. 7), a negative regulator of ABA signaling. These findings suggest that over-expression of *IbSUT4* enhanced the ABA response, by activating the ABA receptors and positive regulators and inhibiting the negative regulator.

## Discussion

Sucrose is produced in source leaves during photosynthesis and functions as a carbon source. It is transported throughout the plant by SUTs, where it regulates development and adaptation to environmental challenges. Thus, SUTs play an undeniably important role in plant growth and stress tolerance [34–37]. A more complete understanding of the mechanisms by which SUTs work can better enable the modulation of sucrose transport for better yield and improved quality in the sweetpotato. In this study, *IbSUT4–*a conserved SUT gene whose encoded protein is localized in the tonoplast and plasma membrane and is highly expressed in sink organs – was studied to investigate its roles in plant growth and resistance to abiotic stress. The results indicated that the over-expression of *IbSUT4* in *Arabidopsis thaliana* might improve sucrose efflux from source leaves, thereby increasing sucrose transport from source to sink organs, resulting in sucrose accumulation in the roots, early flowering, and improved resistance to abiotic stress through the ABF-dependent ABA signaling pathway. Our study highlights the importance of *IbSUT4* in plant growth and the plant’s response to abiotic stress.

### *IbSUT4* encodes a functional protein and supports sucrose transport

The best characterized function of the sucrose transporters in the plants is the uptake of sucrose into the phloem for long-distance photoassimilates transport. However, it has been reported that some SUTs, such as *AtSUC3* and *LeSUT4*, can not transport sucrose [38]. A previous study showed that *AtSUC6* was a pseudogenes coding for non-functional proteins [39]. More recent work has shown that *AtSUC6* is a high-affinity H^+_^ symporter mediating sucrose uptake [40]. The evolutionary distances between plants and yeast mean that the plant proteins may not target the correct membrane in yeast [41, 42], or that they lack the essential protein modifications and do not show normal activity in a heterologous system. Here, we used the SUSY7/Ura3 yeast strain transformed with *IbSUT4* to verify sucrose uptake activity in *IbSUT4*. The transformed cells grew better (Fig. 3a and Fig. 3b), which was consistent with the observations of *StSUT1* and *AtSUT4* grown on media containing sucrose as the sole carbon source [10, 43]. Esculin is a fluorescent analog of sucrose that can be transported in plants and easily observed. *Arabidopsis thaliana* and tomato plants with *MdSUT2* over-expression showed more intense esculin fluorescence in the root tips, indicating the sucrose uptake of *MdSUT2*, and suggesting enhanced activity in the *MdSUT2* lines [44]. The esculin fluorescence intensity was stronger in the transgenic lines than in the WT plants (Fig. 3d). The intensity of esculin fluorescence in the transgenic *Arabidopsis* root tips, and their outward expansion to accumulate esculin, may verify the sucrose uptake function of *IbSUT4* and explain the sequestration of esculin into the parenchymal cell vacuoles.

We present several lines of evidence to support the hypothesis that *IbSUT4* mediates the source-sink distribution of sucrose and the sucrose efflux from leaves and vacuoles in the sweetpotato. *IbSUT4* was localized in the tonoplast and plasma membrane (Fig. 2), where it was involved in sucrose transport across the membranes. Furthermore, the transcription level of *IbSUT4* was higher in the sink tissues than in the source tissues (Fig. S1C). In dicot annuals, SUTs such as *AtSUC4* are predominantly expressed in the anthers, shoot apical, and floral meristems, and the genes of the sink organs belong to clade IV [10, 45]. This expression pattern is consistent with our observations of *IbSUT4* in the root stele. *AtSUC4* was verified to release sucrose from the *Arabidopsis thaliana* vacuoles [42]. Sinks organs are not carbon autonomous but depend on the supply of photoassimilates from the source organs. Sucrose is essential for sink metabolism and supports growth and development. Therefore, sinks retain their vacuolar sucrose at low concentrations and may use clade IV sucrose transporters to re-export sucrose. We also determined the sucrose content of the shoots and roots of WT and transgenic lines. Over-expression of *IbSUT4* in *Arabidopsis thaliana* resulted in higher sucrose accumulation in roots than in those of the WT (Fig. 6), suggesting that *IbSUT4* enhances sucrose efflux from the leaves and promote sucrose distribution to the sink organs. Payyavula et al. (2015) suggested that silencing *PtaSUT4* reduced sucrose efflux from source leaves, based on observations of sucrose reduction in importing YL (young leaf) and ST (shoot tip) [12]. We hypothesized that *IbSUT4* regulates the release of sucrose stored in the root vacuoles during root growth and development. When this sucrose is consumed, low cytosolic concentrations promote sucrose efflux from the source leaves. Future studies should develop transgenic *IbSUT4* sweetpotato lines to verify this pathway.

### *IbSUT4* plays important roles in plant growth and resistance to abiotic stress

In addition to being transported from the source to the sink as the main carbohydrate, sucrose may also serve as a non-nutritional regulator of cellular metabolism by altering gene expression [46]. Therefore, SUTs play a vital role in plant growth and the response to abiotic stress. In higher plants, flowering signifies the transition from the vegetative to the reproductive phase and is important for plant growth and development. It is controlled by many transduction pathways, including the photoperiod-dependent phytochrome signal transduction pathway, the sucrose pathway, and the GA_3_ pathway [47–49]. *FT* encodes a protein similar to the phosphatidylethanolamine-binding protein and promotes flowering under LD conditions [50, 51]. In this study, we observed early flowering and increased *AtFT* expression in the *IbSUT4* expressing lines (Fig. 4). This suggests that *IbSUT4* is involved in plant growth and development.

Previous studies have shown significantly lower MDA content in three *MdSUT2* over-expression lines (MdSUT2/Col-1/2/3) under stress treatment, indicating that *MdSUT2* plays important roles in resistance to abiotic stress, and this was further supported by the root lengths analysis [44]. The rate of the superoxide anion production increases with improved stress intensity, causing an imbalance in the metabolism of superoxide anions in the cell. This results in excess superoxide anions that induce a free radical chain reaction, generating more free radicals and reactive oxygen species, which oxidizes the double bonds of unsaturated fatty acids in the membrane cells, leading to break down and destruction. MDA is an important product of lipid peroxidation in the cell membranes, whose concentration varies in response to biotic and abiotic stressors [52]. Here, we found that less MDA was generated in the *IbSUT4* transgenic *Arabidopsis thaliana* seedlings (Fig. 5d), indicating that over-expression of *IbSUT4* can significantly improve plant tolerance to stress conditions.

Generally, environmental stress affects sucrose supply by inhibiting photosynthesis. Therefore, the trans-membrane transport and distribution of sucrose in plants is a key step in plant stress resistance. To date, the cumulative evidence suggests that SUTs influence the plant stress response by altering the sucrose allocation from the source to sink organs*. PtaSUT4*-RNAi caused substantial sucrose accumulation in the vacuole and affected the water balance to such an extreme that *PtaSUT4*-RNAi plants wilt in short-term drought conditions [14]. Loss-of-function mutation in *AtSUC2* and *AtSUC4* led to hypersensitive responses to abiotic stress and ABA treatment during seed germination and seedling growth and higher sucrose levels in the shoots [13]. High sucrose content in the shoots and low sucrose content in the roots were observed in the *AtSUC4* mutant, under drought and salt stress [53]. The over-expression of *IbSUT4* caused a greater accumulation of sucrose in the roots and a lower accumulation in the shoots, under abiotic stress conditions (Fig. 6). The transgenic *IbSUT4* lines responded to abiotic stress by altering their sucrose allocation, highlighting the potential of *IbSUT4* in the stress response.

### *IbSUT4* responds to abiotic stress through the ABF-dependent ABA signaling pathway

The plant hormone ABA is involved in many processes of plant growth and development, including inhibiting seed germination, maintaining seed dormancy, controlling stomatal closure, and the adaptive responses to environmental stress [54, 55]. Adaptation to abiotic stress is regulated by ABA-dependent and ABA-independent pathways [56, 57]. Disruption of *AtSUC2*, *AtSUC4*, and *AtSUC9* results in hypersensitive responses to abiotic stress, and ABA treatment during seed germination and seedling growth inhibits the expression of ABA-induced and ABA-responsive genes [13, 23], suggesting that *AtSUC2*, *AtSUC4,* and *AtSUC9* are involved in the abiotic stress response through the ABA-dependent signaling pathway. The ABA-dependent signaling pathway in plants operates by changing the expression of ABA-regulated genes containing *ABFs*, *SNF1*, and *PP2C*, such as *ABI1*, *ABI2* [18, 58–60]. Here, our promoter analyses confirmed the presence of the ABRE-motif in the *IbSUT4* promoter (Table S3). ABRE is a conserved ABA-responsive *cis*-element that controls ABA-regulated gene expression [18, 61]. *MdAREB2* combined with the ABRE-motif element in the *MdSUT2* promoter regulated the stress-induced response of sucrose accumulation via ABA-signaling pathway [33]. Further analyses of the ABA-regulated gene expression profiles showed that ABFs (*ABF2, ABF4*), ABFs upstream (*SnRK2.2, SnRK2.3*), and ABA receptor (*PYL8/RCAR3*) were morer highly expressed in the ABA treatment, except ABF downstream (*ABI1*) (Fig. 7). Several instances of cross-talk between the stress and ABA signaling pathways involved SnRKs (sucrose non-fermenting-related protein kinase), which are key metabolic regulators of the stress response [61]. Over-expression of *SnRK2C* in *Arabidopsis thaliana* improved the expression of stress response genes during drought tolerance [19]. *ABI1* (ABA insensitive 1), a member of the PP2C gene family, is a negative regulator of ABA signal transduction. *BcABI1* is a highly homologous gene to *AtABI1* that presents a constitutive expression pattern and participates in wound stress and high salt responses [62]. Thus, we speculate that *IbAREBs* combine with the ABRE-motif in the *IbSUT4* promoter to respond to stress through the ABF-dependent ABA signaling pathway.

## Conclusions

**Fig. 8:**
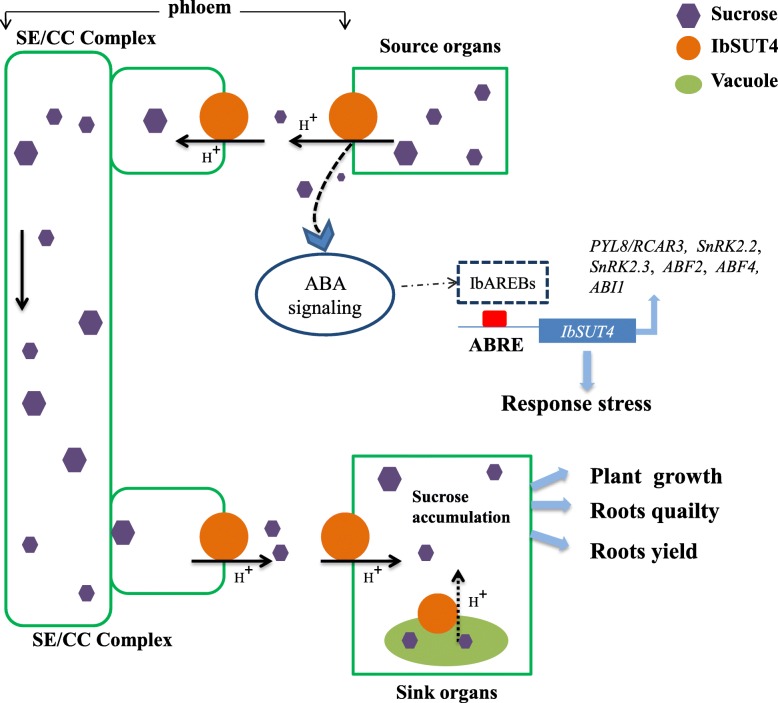
Schematic illustration of *IbSUT4* mediating sucrose transport during plant growth, development, and in response to stress. Normally, the sucrose from source cells is transported by SUTs to the root parenchyma cells via the apoplast pathway. Over-expression of *IbSUT4* may promote sucrose efflux from the source leaves, leading to increased sucrose distributions from source to sink tissues and improved sucrose content in the roots, which ultimately leads to better plant growth, improves quality, and increases yield. Under stress, *IbSUT4-*mediated sugar signaling and ABA signaling may converge and cross-talk through specific factors (such as IbAREBS) to affect the expression of ABA signaling pathway genes and the allocation of sucrose. SE/CC: Sieve companion

In summary, *IbSUT4* is an important SUT gene, encoding a protein that is localized in the tonoplast and plasma membrane. It also influences the sucrose distribution between source and sink tissues. Over-expression of this gene accelerated *Arabidopsis thaliana* growth and improved abiotic stress resistance by enhancing the sucrose content of the sink organs. Our study reveals the critical role of *IbSUT4* in plant growth and abiotic stress response. The function of SUTs is highly conserved, so the accumulation of sucrose in *Arabidopsis* roots suggests that *IbSUT4* influences the quality and yield of root crops, especially sweetpotato (Fig. 8). Future studies should verify these functions by developing transgenic sweetpotato lines. A more complete understanding of how *IbSUT4* affects the sucrose distribution of plants may open new avenues for improving crop yield.

## Methods

### Plant materials and growth conditions

These *Arabidopsis thaliana* and tobacco plants used in this study were obtained in a climate chamber at the Institute of Plant Physiology and Ecology, Chinese Academy of Sciences, Shanghai (31°18′N, 121°43′E)*.* The *Ipomoea batatas “*Taizhong 6*”* was cultivated in the field at the Wushe Plantation for Transgenic Crops, Shanghai (31°14′N, 121°29′E).

*Arabidopsis thaliana* (Columbia ecotype, Col-0), used for experiments, were maintained under LD conditions, at approximately 600 mmolm^− 2^ s^− 1^, at 25 °C. The seed germination experiments and stress tolerance assays were performed according to the method described by Mas [44], with some modifications. We added a 10 °C processing. For the plant phenotype analyses, WT and transgenic plants were germinated on the MS medium for 10 d and then grown in individual pots in a greenhouse under LD conditions, with 6 replicates. For the analyses of the ABA signaling pathway genes, WT and transgenic plants were grown on 1/2 MS medium for 14 d, and then treated with 40 μM ABA for 6 h.

The tobacco (*Nicotiana benthamiana* Domin; all in this lab) used for the subcellular localization assays were grown in the greenhouse, in potting soil, and under LD conditions.

Sweetpotato (*Ipomoea batatas* (L.) Lam; all in this lab) “TaiZhong 6”, used for gene expression analyses, was grown in Shanghai, in a Wushe farm. Tissues were sampled from 3 month-old sweetpotato plants.

### Cloning of the sucrose transporter cDNAs

Approximately 100 mg of sweetpotato source leaves were ground with liquid nitrogen and transferred to a 1.5 mL centrifuge tube. Total RNAs were extracted from the source leaves using RNAprep Pure Plant Kit (TIANGEN, China). E4-F and E4-R primers (Table S1) were designed according to the conserved sequences of plant SUT genes, and the EST fragment of *IbSUT4* gene was amplified and sequenced.

To acquire the full-length SUT cDNA sequences, two rounds of nested rapid amplification of cDNA ends (RACE) PCR techniques were performed. SpSUT4F/R primers (Table S1) were designed according to the EST sequences, and the 5′ and 3′ ends of *IbSUT4* cDNA were amplified and sequenced, using SMART RACE cDNA Amplification Kit (Clontech, Japan), according to the manufacturer’s instructions. The full-length *IbSUT4* sequence was obtained by splicing sequences, and the *IbSUT4*-F/R primers (Table S1) were designed according to the full-length sequences, which was then amplified using cDNA as the template and ReverTra Ace qPCR RT Master Mix’s (TOYOBO, Japan), according to the manufacturer’s instructions.

### Sequence alignment and phylogenetic tree construction

To identify the homologs of *IbSUT4*, BlastP searches using the full-length *IbSUT4* protein sequences were performed against the sequences of *Arabidopsis thaliana, Nicotiana tabacum, Solanum tuberosum, Solanum lycopersicum, Malus domestica,* in the National Center for Biotechnology Information (NCBI). Protein sequences were aligned using MEGA5.2, and unrooted phylogenetic trees were constructed using the Neighbor-Joining method in MEGA5.2. The reliability of the trees was estimated with 1000 bootstrap iterations.

### Subcellular localization analyses and esculin uptake assay

To determine the subcellular localization of IbSUT4 in sweetpotato, ORFs, without the stop codon, were amplified by PCR (Table S1)*.* The *IbSUT4* amplified product was digested with SalI/SpeI and then cloned into the region between the CaMV35S promoter and GFP of the PUC18 binary vector. The resulting CaMV35S:IbSUT4-GFP (PUC18) construct was used to transform tobacco protoplasts. Leaf mesophyll protoplasts from two-month-old tobacco plants were generated as described by Drechsels [63]. After transformation via the polyethylene glycol method [64]. Protoplasts were kept in tubes for approximately 18 h at 22 °C in darkness. PI in W5 buffer was added to 500 μL of protoplasts suspension in W5 solution (pH = 5.7) in a 1.5 mL tube to a final concentration of 50 μg mL^− 1^. The tubes were gently inverted, and the protoplasts were incubated for 30 min at room temperature in darkness. The solution was centrifuged and washed with W5 solution after discarding the supernatant.

Esculin uptake by the seedlings was assessed according to the methods described by Ma QiJuns [44]. Quantitative analyses of the fluorescence intensity were conducted in Image J software by calculating the average optical density; the value of IntDen / Area of three pictures.

Protoplasts and *Arabidopsis thaliana* were observed using an Olympus FV1000 confocal laser-scanning microscope, equipped with a 603/1.2 NA UPLSAPO oil immersion objective lens (Olympus). GFP fluorescence was observed using excitation and emission wavelengths of 473 nm and 487–521 nm, respectively. Chloroplast autofluorescence was observed by excitation and emission at 559 nm and 606–673 nm, respectively. PI fluorescence was observed by excitation and emission at 535 nm and 615 nm, respectively. Esculin fluorescence was observed by excitation and emission at 367 nm and 454 nm, respectively.

### Functional analysis of *IbSUT4* in yeast SUSY7/ura3

The yeast mutant SUSY7/ura3 can not synthesize invertase [4]. It can metabolize sucrose in the cell and grow normally on the media containing sucrose as the sole carbon source, when the gene encoding the sucrose synthase in the plant is tranferred into SUSY7/ura3. Heterologous expression and complementation of sucrose in the defective yeast is direct envidence to verify the sucrose transport activity of the SUT genes. We cloned the *IbSUT4* ORF by PCR (Table S1). The *IbSUT4* ORF was excised with ClaI/SmaI, and then cloned into the same sites of p416GPD. To analyze the SUT activity, the vector (*IbSUT4*-P416) was transferred into SUSY7/ura3 competent cells. Large, healthy colonies from the bait and control strains were selected and resuspend individually in 0.9% NaCl, and the OD600 was adjusted to ~ 1. Each transformation reaction was then diluted 10, 100, 1000, and 10,000 times. Ten microliters of each dilution reaction were plated on an agar medium containing 1.7 g L^− 1^ yeast nitrogen base without amino acids (Difco), 2% sucrose (Difco) or 2% glucose (Difco), 5 g L^− 1^ ammonium sulfate, 20 mg L^− 1^ tryptophan, and 1.5% agarose, and the pH was adjusted to 5.0 using HCl. The plates were maintained at 30 °C for 3 d and then observed and photographed.

### Amplification of the *IbSUT4* promoter

To analyze the promoter of the *IbSUT4*, we amplified the promoter by genome walking method. Primers were designed from known DNA sequences in the inverse direction. The reverse primer was designed from the 5′ end, and the forward primer was designed from the 3′ end. Primers were located at 100–200 bp away from the ends (Table S1). First, 5 μg genomic DNA was digested with a restriction enzyme that does not uncut between the primers. The digestion reaction was carried out at 37 °C for 6–8 h. The digested DNA was purified with an equal volume of phenol/chloroform, centrifuged at 10,000 g at 4 °C for 10 min, and the upper phase was collected. An equal volume of chloroform/isoamyl alcohol mixture was further added, the solution was vortexed and centrifuged, and the upper phase was collected. The DNA was precipitated with 0.1volume 3 M sodium acetate and 2.5 volume 100% ethanol and centrifuged, and the supernatant was discarded. The pellet was washed with 80% ethanol and centrifuged, and the supernatant was discarded. The pellet was air-dried and then resuspended in water, and the T4 enzyme was used for ligation. These steps were repeated to precipitate and depurated the DNA, and two rounds of PCR were performed to amplify the promoter.

### Transformation of *IbSUT4* into *Arabidopsis*

The full-length coding sequence of *IbSUT4* was amplified by PCR and inserted into the pCAMBIA1301 vector. The resulting 35S:*IbSUT4* construct was introduced into the *Agrobacterium* strain GV3101 for *Arabidopsis thaliana* transformation using the floral dip method [65]. The seeds were collected and sown on 1/2 MS medium containing 25 mg L^− 1^ hygromycin for selection.

### qRT-PCR assays

Gene expression was quantified with specific qRT-F and qRT-R primers. Actin was used as the control. The primers used for assays are shown in Table S1. Each sample was assayed in three biological replicates. The relative quantification of specific mRNA levels was performed using the cycle threshold (Ct) 2^-DDCt^ method [66].

### Determination of sucrose content and malondialdehyde (MDA) content

The sucrose concentrations were determined by high-performance liquid chromatography (HPLC), as described by Gongs [13].

The MDA content was determined according to the method described by Mas [44], with some modifications. We used 10% TCA to extract MDA from the roots.

## Supplementary information

**Additional file 1: Table S1.** Sequences of the primers used in this study **Table S2.** Count the number of flowering plants. **Table S3.** Cis-element of the *IbSUT4* promoter. **Figure S1.** A. EST sequences. B. The 5′-RACE and 3′-RACE amplification of *IbSUT4*. C. Expression of *IbSUT4* gene was assessed by qRT–PCR in the different tissues of “Taizhong 6” sweetpotato. Three-month-old samples were collected from the field. DL: development leaf, B: handle, S: stem, WR: white root, RR: red root, DR: development root, MR: mature root. (A, B) The gels are cropped from Additional file 2, Figure S.1. Lowercase letters indicate statistically significant differences (*P* ≤ 0.05). **Figure S2.** In vivo sucrose uptake activity of *IbSUT4*. The average optical density was significantly higher in the roots of transgenic plants than in the WT plants. Average optical density = IntDen/Area. Lowercase letters indicate statistically significant differences (*P* ≤ 0.05). **Figure S3.** The expression of *IbSUT4* in the roots and leaves of one-month-old plant treated with low temperature (4 °C), high salt (200 mM NaCl), drought stress (300 mM Mannitol), or exogenous ABA (25 μM). A. Quantification of *IbSUT4* expression in the leaves. B. Quantification of *IbSUT4* expression in the roots. Lowercase letters indicate statistically significant differences (*P* ≤ 0.05).

**Additional file 2 Figure S1.** The original and full-length gels of EST, 5′-RACE and 3′-RACE. A. EST4 sequences. B. The 5′-RACE and 3′-RACE amplification of *IbSUT4*. **Figure S2**. The original and full-length gel of RT-PCR analysis of *IbSUT4* expression in the WT and three transgenic lines. **Figure S3.** The original blot of *IbSUT4* function analysis in the SUSY7/ura3 yeast strain. A. SUSY7/ura3 yeast transformed with the empty P416 vector and with *IbSUT4* in the P416 vector, and grown on 2% sucrose. B. SUSY7/ura3 yeast transformed with the empty P416 vector and with *IbSUT4* in the P416 vector, and grown on 2% glucose.

## Data Availability

The datasets supporting the conclusions of this article are included within the article and its additional files. About genes database could download from NCBI by their accession number. The accession numbers of these genes are as follows: *AtFT* (At1g65480), *AtABI3*(At3g24650), *ABF2* (ABO17160), *ABF4* (At3g19290), *SnRK2.2* (AEE78673), *SnRK2.3* (NP201489), *PYL8/RCAR3* (At5g53160) and *ABI1* (At4g26080), *IbSUT1* (ADK62516.1), *IbSUT2* (ADL63117.1), *IbSUT3* (MN233361), *IbSUT4* (MN233360), *LeSUT1* (CAA57726.1), *LeSUT2* (AAG12987.1), *LeSUT4* (AAG09270.1), *StSUT1* (CAA48915.1), *StSUT2* (AAP43631.1), *StSUT4* (AAG25923.2), *NtSUT1* (CAQ58421.1), *NtSUT3* (AAD34610.1), *NtSUT4* (BAI60050.1), *AtSUC1* (AAV97807.1), *AtSUC2* (AAN31829.1), *AtSUC4* (AAO42412.1), *AtSUC5* (NP177334.1), *AtSUC7* (NP001031242.1), *AtSUC8* (AEC06318.1), *AtSUC9* (NP196235.1), *MdSUT4* (NP001280809).

## Abbreviations

ABA: Abscisic acid
ABFs: ABA-responsive element binding factors
B: Handle
DL: Development leaf
DR: Development root
HPLC: High-performance liquid chromatography
LD: Long Day (16 h light / 8 h dark)
MDA: Malondialdehyde
MFS: Major Facilitator Superfamily
MR: Mature root
MS: Murashige and Skoog
NCBI: National Center for Biotechnology Information
ORF: Open reading frame
PI: Propidium Iodide
qRT-PCR: Quantitative reverse- transcription-PCR
RACE: Rapid Amplification of cDNA Ends
RR: Red root
S: Stem
SE-CCs: Sieve element-companion cells
ST: Shoot tip
SUTs or SUCs: Sucrose transporters or carriers
TCA: Trichloroacetic acid
WT: Wild-type
WR: White root
YL: Young leaf
