# Supplementary material for: A novel sucrose transporter gene IbSUT4 involves in plant growth and response to abiotic stress through the ABF-dependent ABA signaling pathway in Sweetpotato
Source: BMC Plant Biol. 2020 Apr 15;20:157. doi: 10.1186/s12870-020-02382-8 (PMC7157994; doi:10.1186/s12870-020-02382-8)
Supplement: Supplementary file 1 — Additional file 1: Table S1. Sequences of the primers used in this study Table S2. Count the number of flowering plants. Table S3. Cis-element of the IbSUT4 promoter. Figure S1. A. EST sequences. B. The 5′-RACE and 3′-RACE amplification of IbSUT4. C. Expression of IbSUT4 gene was assessed by qRT–PCR in the different tissues of “Taizhong 6” sweetpotato. Three-month-old samples were collected from the field. DL: development leaf, B: handle, S: stem, WR: white root, RR: red root, DR: development root, MR: mature root. (A, B) The gels are cropped from Additional file 2, Figure S.1. Lowercase letters indicate statistically significant differences (P ≤ 0.05). Figure S2. In vivo sucrose uptake activity of IbSUT4. The average optical density was significantly higher in the roots of transgenic plants than in the WT plants. Average optical density = IntDen/Area. Lowercase letters indicate statistically significant differences (P ≤ 0.05). Figure S3. The expression of IbSUT4 in the roots and leaves of one-month-old plant treated with low temperature (4 °C), high salt (200 mM NaCl), drought stress (300 mM Mannitol), or exogenous ABA (25 μM). A. Quantification of IbSUT4 expression in the leaves. B. Quantification of IbSUT4 expression in the roots. Lowercase letters indicate statistically significant differences (P ≤ 0.05). [file 12870_2020_2382_MOESM1_ESM.pptx]

## Slide 1
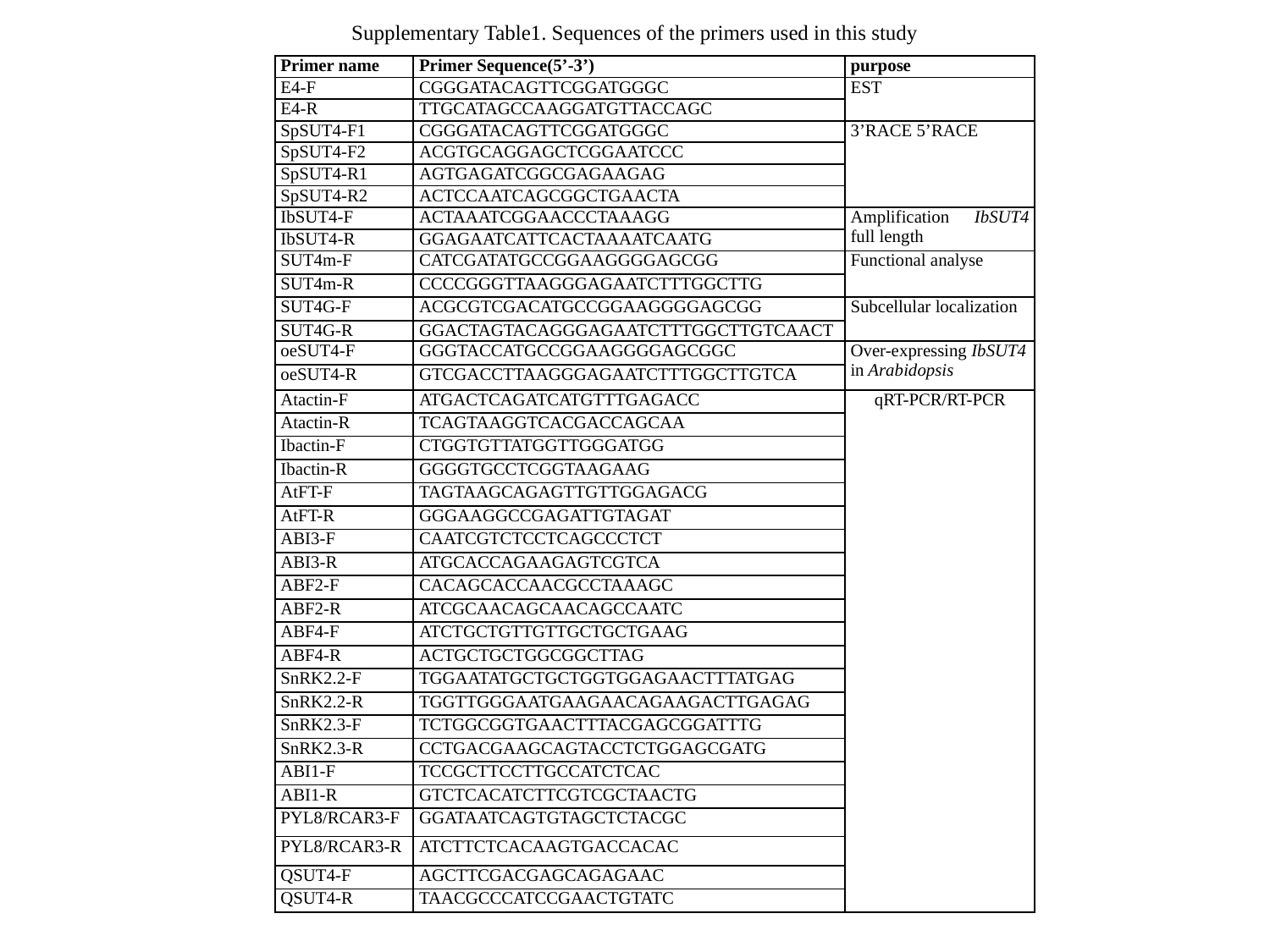

Supplementary Table1. Sequences of the primers used in this study
| Primer name | Primer Sequence(5’-3’) | purpose |
| --- | --- | --- |
| E4-F | CGGGATACAGTTCGGATGGGC | EST |
| E4-R | TTGCATAGCCAAGGATGTTACCAGC | |
| SpSUT4-F1 | CGGGATACAGTTCGGATGGGC | 3’RACE 5’RACE |
| SpSUT4-F2 | ACGTGCAGGAGCTCGGAATCCC | |
| SpSUT4-R1 | AGTGAGATCGGCGAGAAGAG | |
| SpSUT4-R2 | ACTCCAATCAGCGGCTGAACTA | |
| IbSUT4-F | ACTAAATCGGAACCCTAAAGG | Amplification IbSUT4 full length |
| IbSUT4-R | GGAGAATCATTCACTAAAATCAATG | |
| SUT4m-F | CATCGATATGCCGGAAGGGGAGCGG | Functional analyse |
| SUT4m-R | CCCCGGGTTAAGGGAGAATCTTTGGCTTG | |
| SUT4G-F | ACGCGTCGACATGCCGGAAGGGGAGCGG | Subcellular localization |
| SUT4G-R | GGACTAGTACAGGGAGAATCTTTGGCTTGTCAACT | |
| oeSUT4-F | GGGTACCATGCCGGAAGGGGAGCGGC | Over-expressing IbSUT4 in Arabidopsis |
| oeSUT4-R | GTCGACCTTAAGGGAGAATCTTTGGCTTGTCA | |
| Atactin-F | ATGACTCAGATCATGTTTGAGACC | qRT-PCR/RT-PCR |
| Atactin-R | TCAGTAAGGTCACGACCAGCAA | |
| Ibactin-F | CTGGTGTTATGGTTGGGATGG | |
| Ibactin-R | GGGGTGCCTCGGTAAGAAG | |
| AtFT-F | TAGTAAGCAGAGTTGTTGGAGACG | |
| AtFT-R | GGGAAGGCCGAGATTGTAGAT | |
| ABI3-F | CAATCGTCTCCTCAGCCCTCT | |
| ABI3-R | ATGCACCAGAAGAGTCGTCA | |
| ABF2-F | CACAGCACCAACGCCTAAAGC | |
| ABF2-R | ATCGCAACAGCAACAGCCAATC | |
| ABF4-F | ATCTGCTGTTGTTGCTGCTGAAG | |
| ABF4-R | ACTGCTGCTGGCGGCTTAG | |
| SnRK2.2-F | TGGAATATGCTGCTGGTGGAGAACTTTATGAG | |
| SnRK2.2-R | TGGTTGGGAATGAAGAACAGAAGACTTGAGAG | |
| SnRK2.3-F | TCTGGCGGTGAACTTTACGAGCGGATTTG | |
| SnRK2.3-R | CCTGACGAAGCAGTACCTCTGGAGCGATG | |
| ABI1-F | TCCGCTTCCTTGCCATCTCAC | |
| ABI1-R | GTCTCACATCTTCGTCGCTAACTG | |
| PYL8/RCAR3-F | GGATAATCAGTGTAGCTCTACGC | |
| PYL8/RCAR3-R | ATCTTCTCACAAGTGACCACAC | |
| QSUT4-F | AGCTTCGACGAGCAGAGAAC | |
| QSUT4-R | TAACGCCCATCCGAACTGTATC | |

## Slide 2
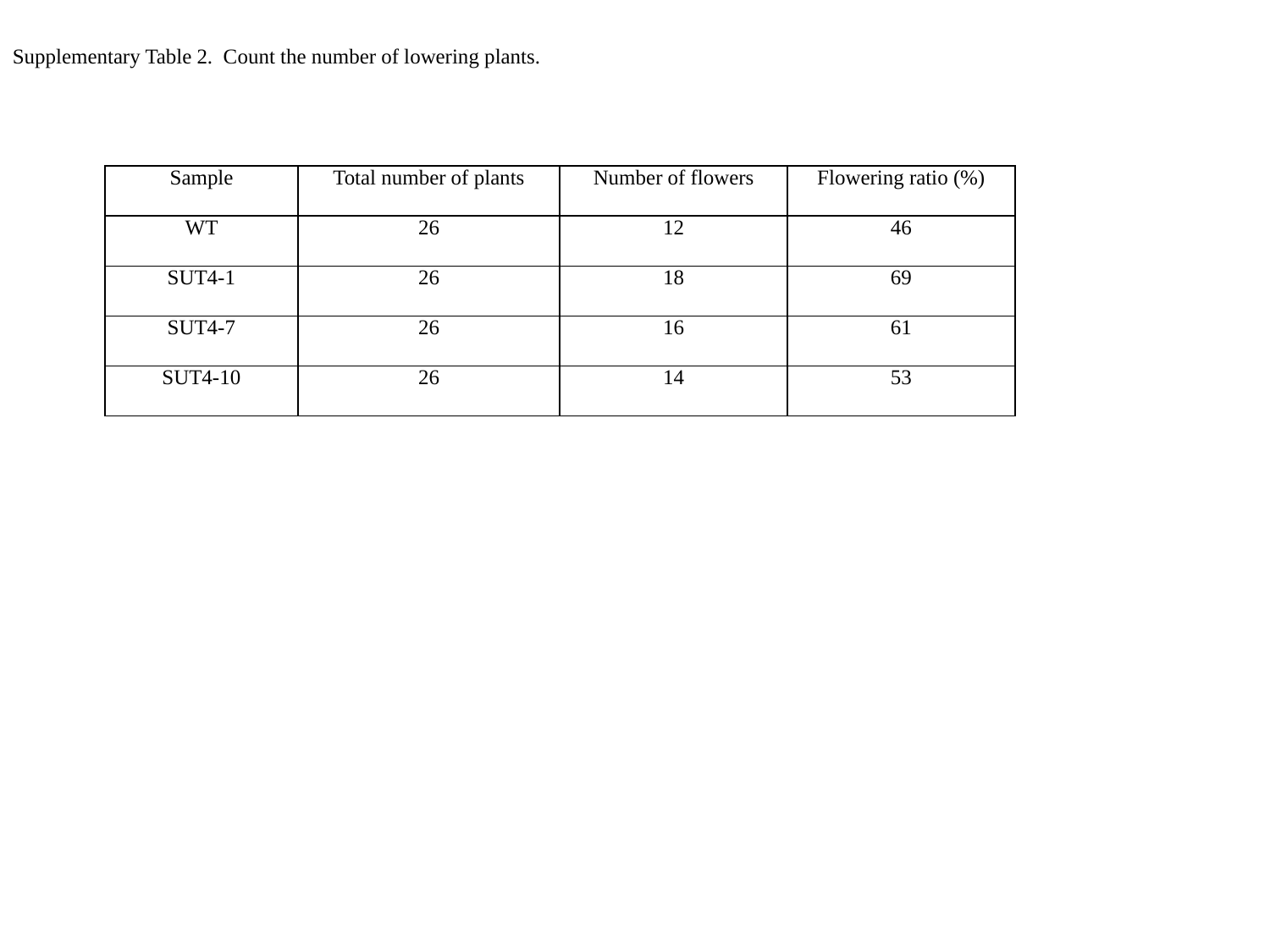

Supplementary Table 2. Count the number of lowering plants.
| Sample | Total number of plants | Number of flowers | Flowering ratio (%) |
| --- | --- | --- | --- |
| WT | 26 | 12 | 46 |
| SUT4-1 | 26 | 18 | 69 |
| SUT4-7 | 26 | 16 | 61 |
| SUT4-10 | 26 | 14 | 53 |

## Slide 3
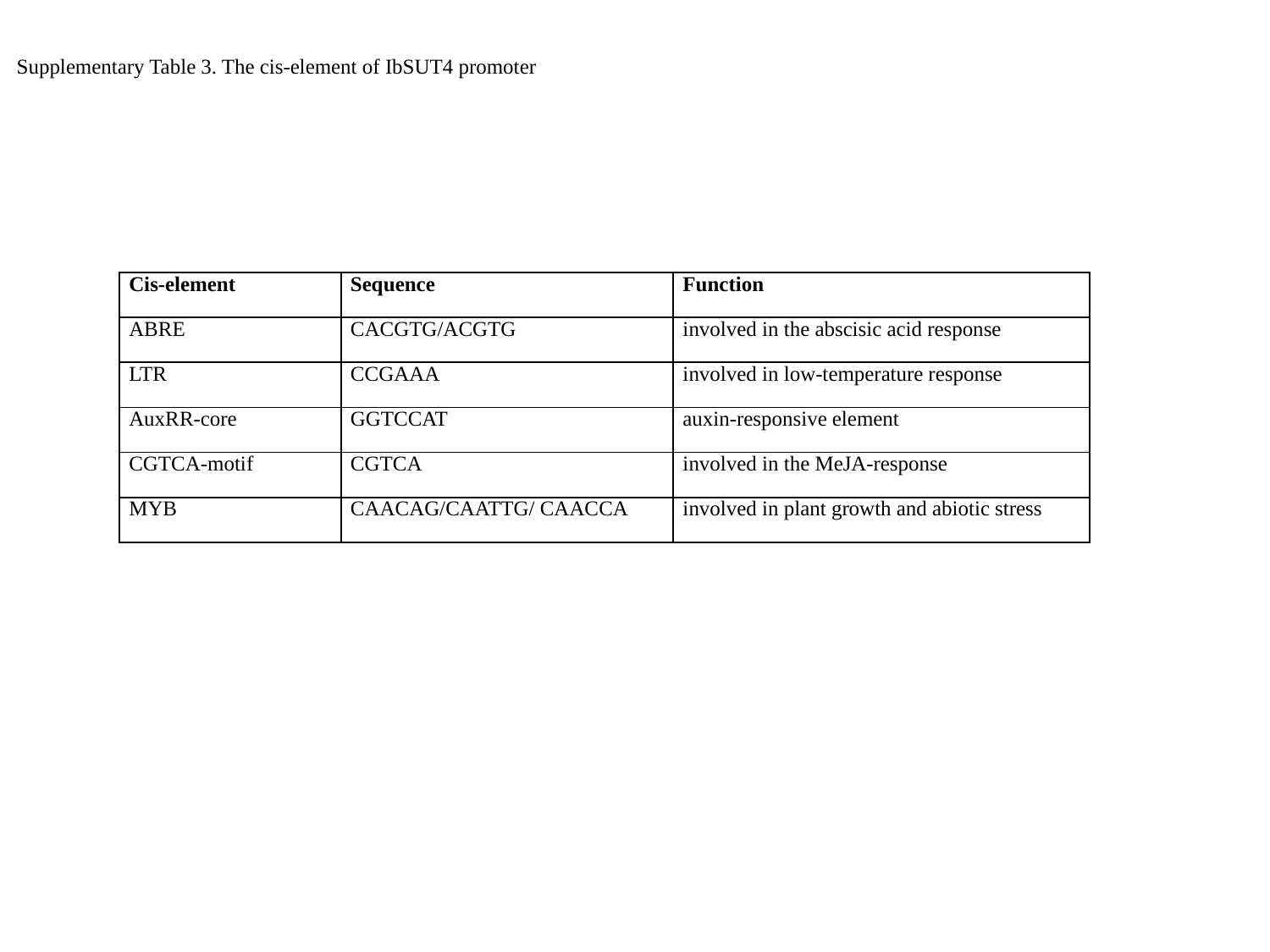

Supplementary Table 3. The cis-element of IbSUT4 promoter
| Cis-element | Sequence | Function |
| --- | --- | --- |
| ABRE | CACGTG/ACGTG | involved in the abscisic acid response |
| LTR | CCGAAA | involved in low-temperature response |
| AuxRR-core | GGTCCAT | auxin-responsive element |
| CGTCA-motif | CGTCA | involved in the MeJA-response |
| MYB | CAACAG/CAATTG/ CAACCA | involved in plant growth and abiotic stress |

## Slide 4
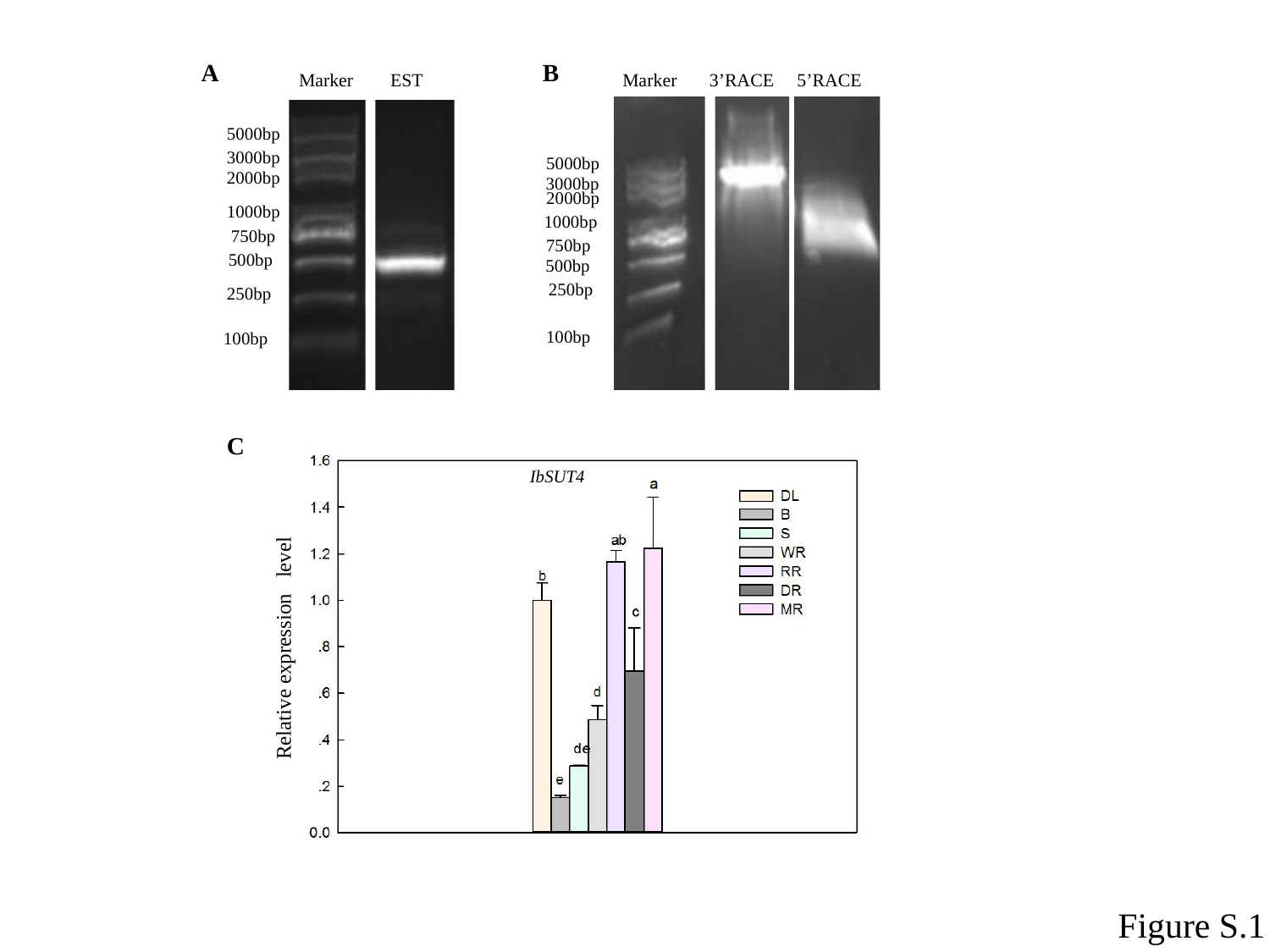

A B
 Marker EST Marker 3’RACE 5’RACE
5000bp
3000bp
5000bp
2000bp
3000bp
2000bp
1000bp
1000bp
750bp
750bp
500bp
500bp
250bp
250bp
100bp
100bp
C
IbSUT4
Relative expression level
Figure S.1

## Slide 5
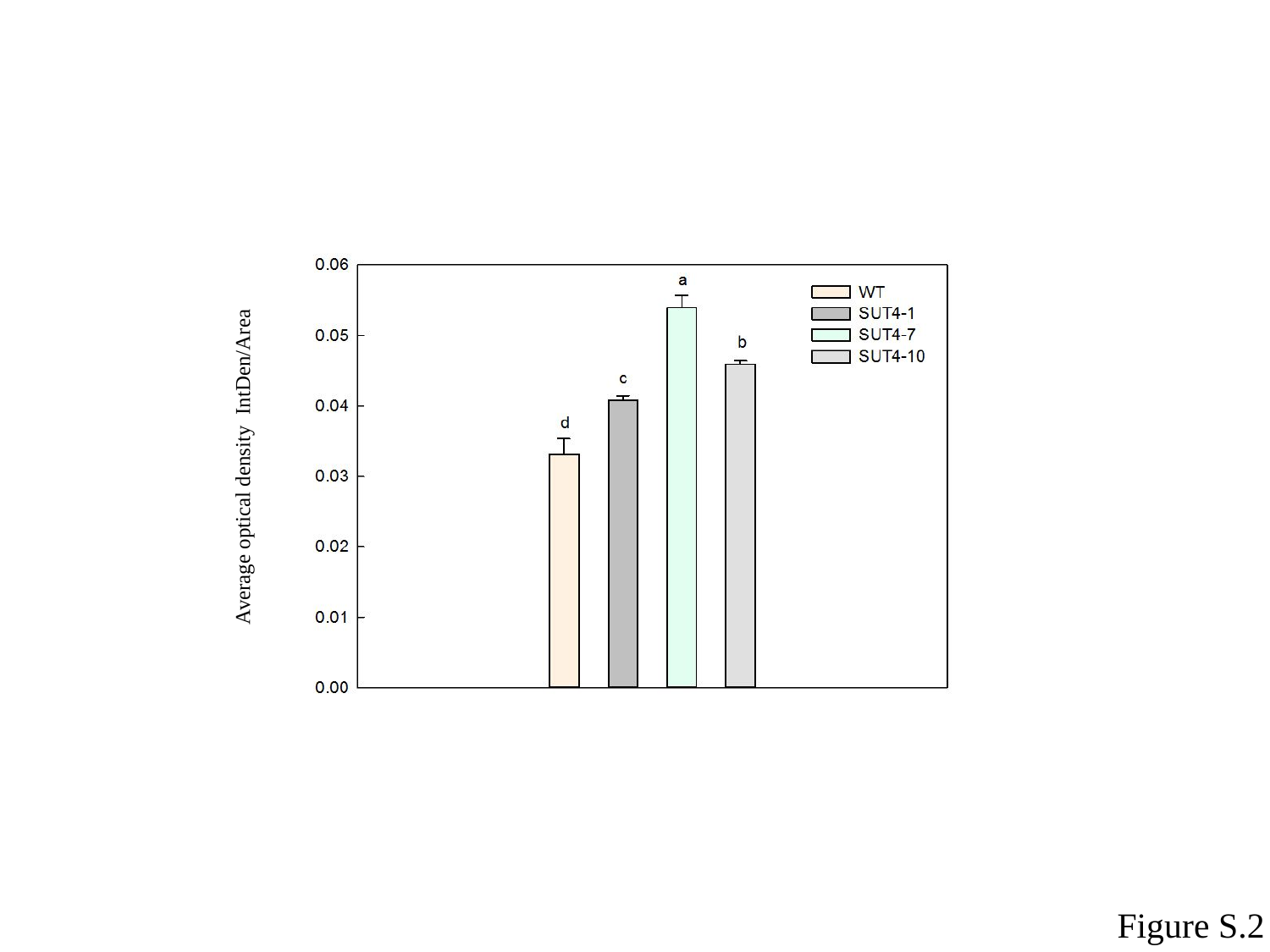

Average optical density IntDen/Area
Figure S.2

## Slide 6
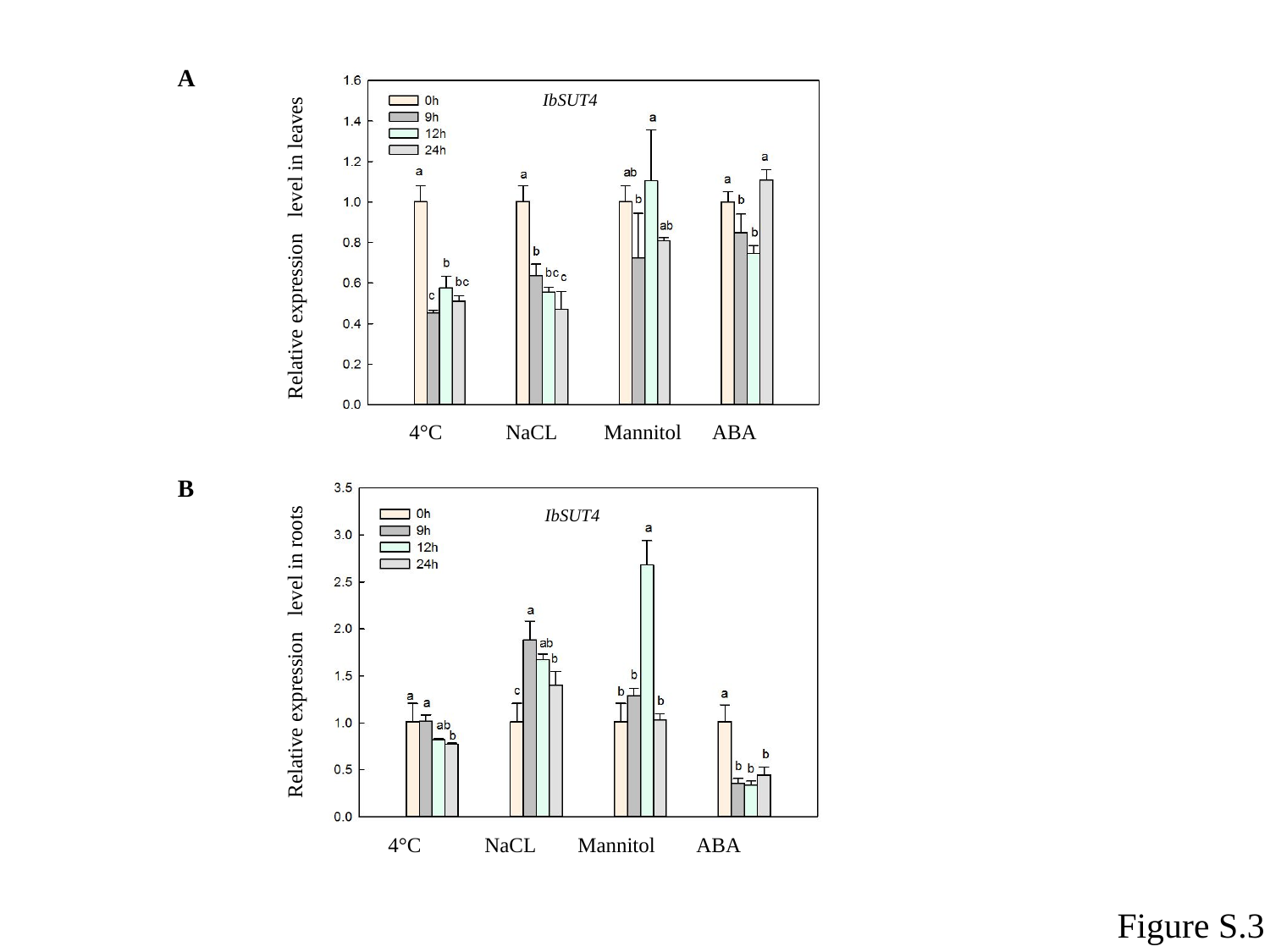

Relative expression level in leaves
A
IbSUT4
Relative expression level in roots
 4°C NaCL Mannitol ABA
B
IbSUT4
 4°C NaCL Mannitol ABA
Figure S.3
