# Supplementary material for: A novel sucrose transporter gene IbSUT4 involves in plant growth and response to abiotic stress through the ABF-dependent ABA signaling pathway in Sweetpotato
Source: BMC Plant Biol. 2020 Apr 15;20:157. doi: 10.1186/s12870-020-02382-8 (PMC7157994; doi:10.1186/s12870-020-02382-8)
Supplement: Supplementary file 2 — Additional file 2 Figure S1. The original and full-length gels of EST, 5′-RACE and 3′-RACE. A. EST4 sequences. B. The 5′-RACE and 3′-RACE amplification of IbSUT4. Figure S2. The original and full-length gel of RT-PCR analysis of IbSUT4 expression in the WT and three transgenic lines. Figure S3. The original blot of IbSUT4 function analysis in the SUSY7/ura3 yeast strain. A. SUSY7/ura3 yeast transformed with the empty P416 vector and with IbSUT4 in the P416 vector, and grown on 2% sucrose. B. SUSY7/ura3 yeast transformed with the empty P416 vector and with IbSUT4 in the P416 vector, and grown on 2% glucose. [file 12870_2020_2382_MOESM2_ESM.pptx]

## Slide 1
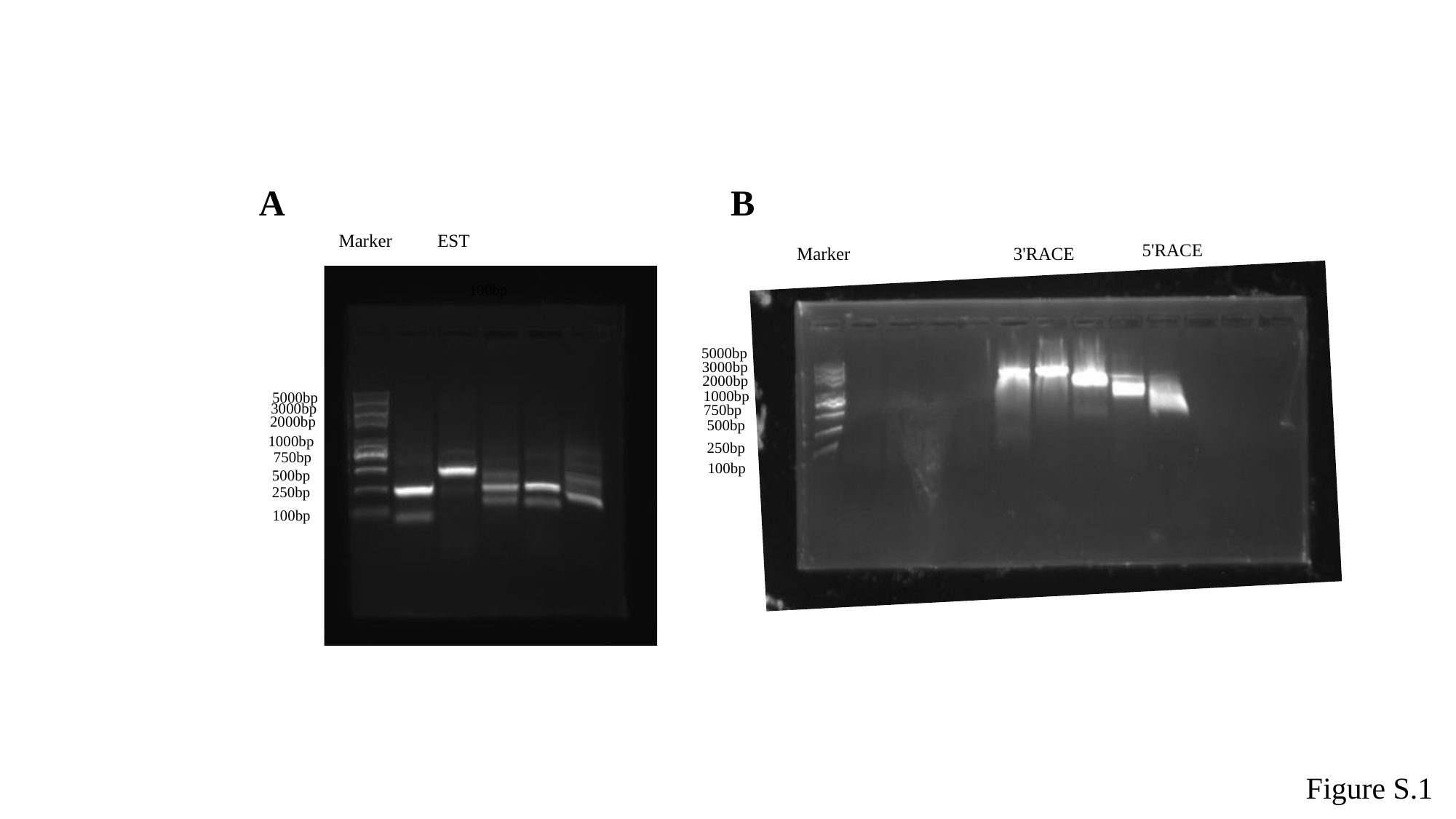

A
B
Marker EST
5'RACE
Marker 3'RACE
100bp
5000bp
3000bp
2000bp
1000bp
5000bp
3000bp
750bp
2000bp
500bp
1000bp
250bp
750bp
100bp
500bp
250bp
100bp
Figure S.1

## Slide 2
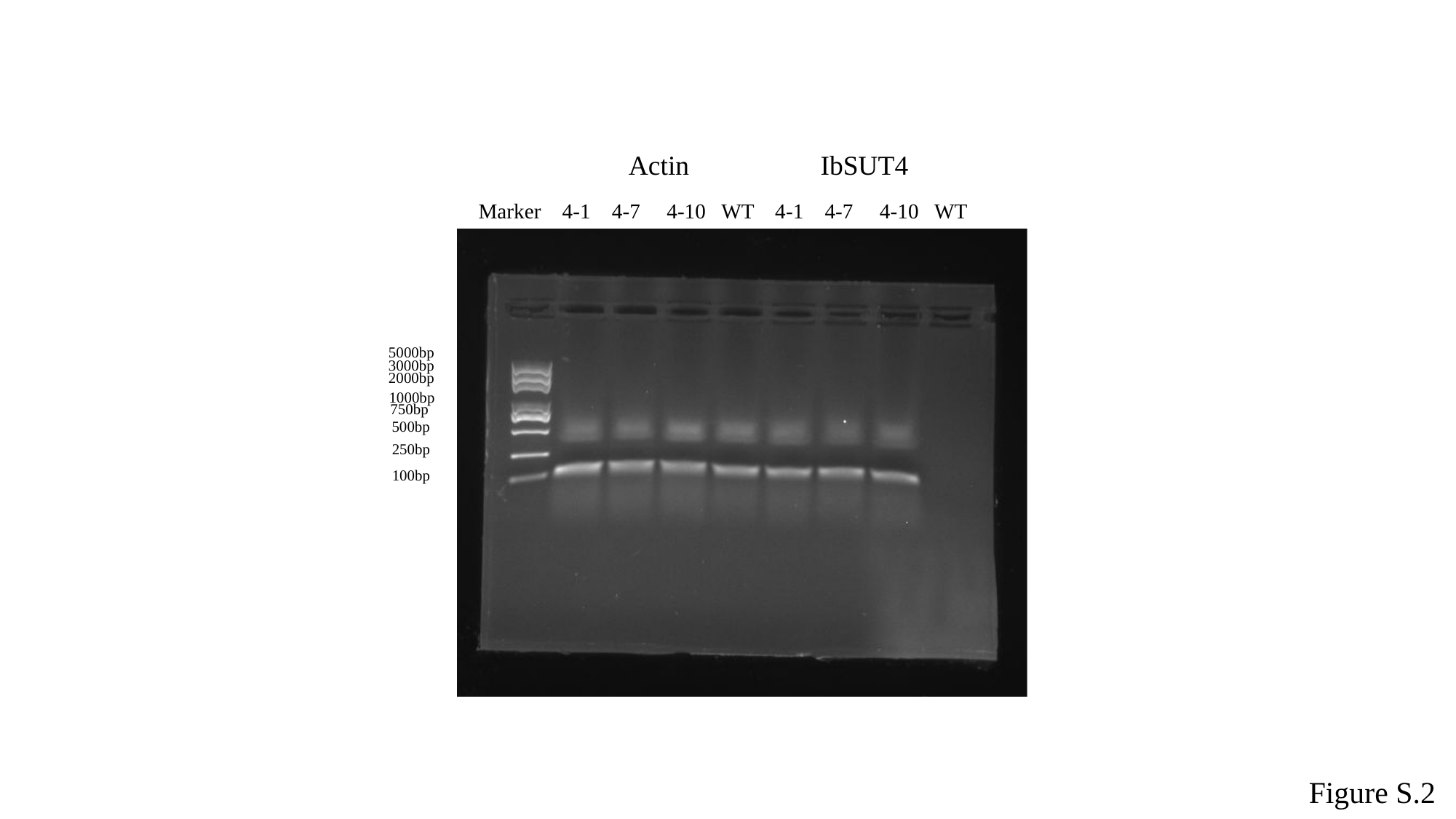

Actin IbSUT4
 Marker 4-1 4-7 4-10 WT 4-1 4-7 4-10 WT
5000bp
3000bp
2000bp
1000bp
750bp
500bp
250bp
100bp
Figure S.2

## Slide 3
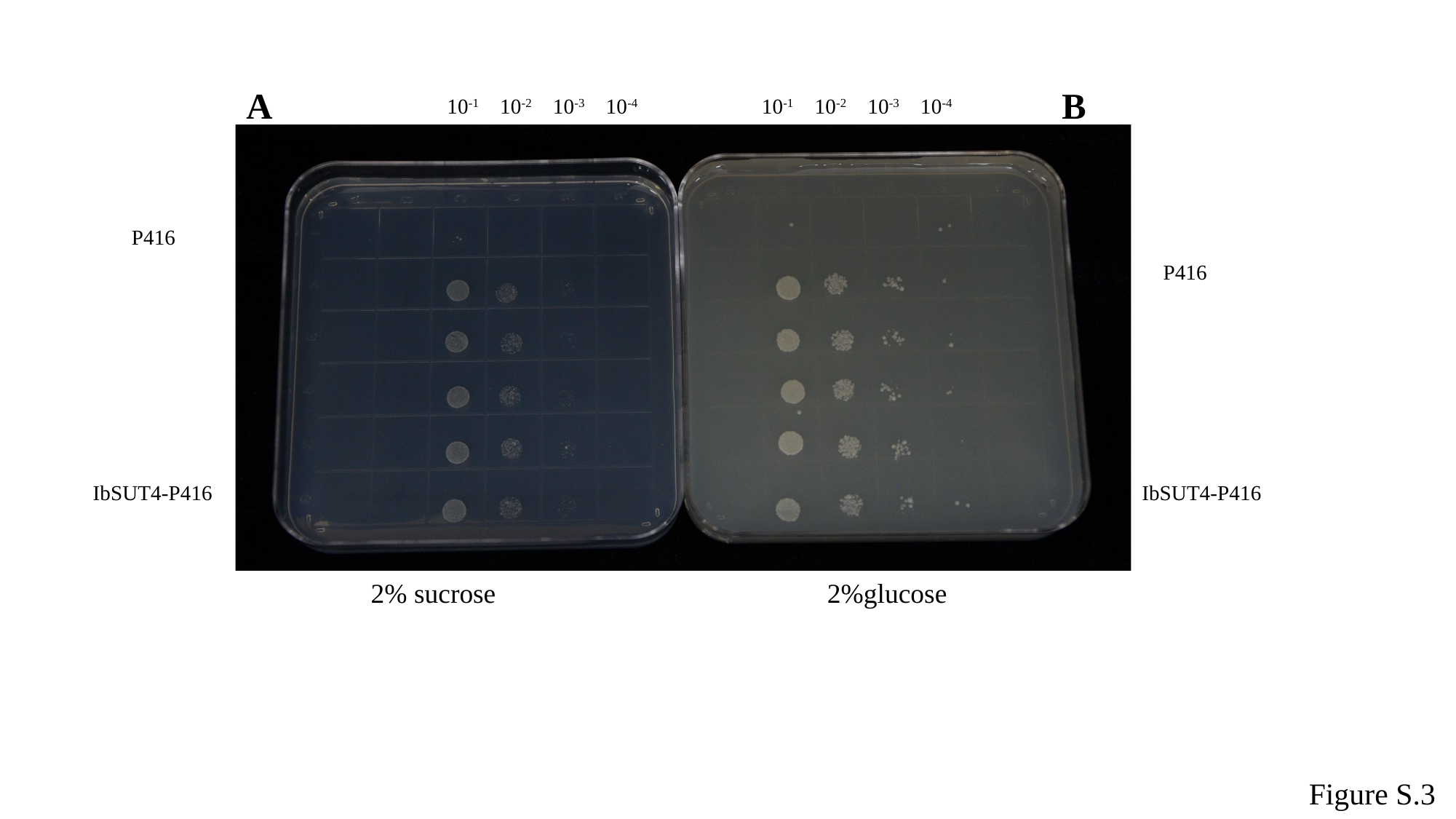

A B
 10-1 10-2 10-3 10-4
 10-1 10-2 10-3 10-4
P416
P416
IbSUT4-P416
IbSUT4-P416
2% sucrose 2%glucose
Figure S.3
